# Female sex workers experiences of using contraceptive methods: a qualitative study in Kenya

**DOI:** 10.1186/s12905-018-0601-5

**Published:** 2018-06-20

**Authors:** Rhoune Ochako, Jerry Okal, Steven Kimetu, Ian Askew, Marleen Temmerman

**Affiliations:** 1 School of Medicine and Health Sciences, Ghent University, Ghent, Belgium; 2 Population Council, Nairobi, Kenya; 3 Department of Reproductive Health and Research, WHO, Geneva, Switzerland; 4 International Centre for Reproductive Health, Ghent University, Ghent, Belgium; 5 Aga Khan University, Nairobi, Kenya

**Keywords:** FSWs, Condom use, Pregnancy prevention, Contraception, Kenya

## Abstract

**Background:**

Female Sex Workers (FSWs) are predisposed to a broad range of social, sexual and reproductive health problems such as sexually transmitted infections (STIs)/HIV, unintended pregnancy, violence, sexual exploitation, stigma and discrimination. Female sex workers have unmet need for contraceptives and require comprehensive Sexual and Reproductive Health (SRH) prevention interventions. Existing programs pay little attention to the broad sexual and reproductive health and rights of these women and often focus on HIV and other STIs prevention, care and treatment while neglecting their reproductive health needs, including access to family planning methods. The aim of this study is, therefore, to explore the experiences of female sex workers with using existing contraceptive methods, assess individual and health facility-level barriers and document inter-partner relationship in the use of contraceptives.

**Methods:**

We focus on women aged 15–49, who reported current sex work, defined as ‘providing sexual services in exchange for money or other material compensation as part of an individual’s livelihood.’

**Results:**

Findings reveal that while some FSWs know about modern contraceptives, others have limited knowledge or out rightly refuse to use contraceptives for fear of losing clients. The interaction with different client types act as a barrier but also provide an opportunity for contraceptive use among FSWs. Most FSWs recognize the importance of dual protection for HIV/STI and pregnancy prevention. However, myths and misconceptions, fear of being tested for HIV at the family planning clinic, wait time, and long queues at the clinics all act in combination to hinder uptake of contraceptives.

**Conclusions:**

We recommend a targeted approach to address the contraceptive needs of FSWs to help remove barriers to contraceptive uptake. We also support the introduction of counseling services to provide information on the benefits of non-barrier contraceptive methods and thereby enhance dual use for both pregnancy and STI/HIV prevention.

## Background

Female Sex Workers (FSWs) are predisposed to a broad range of social, sexual and reproductive health problems such as sexually transmitted infections (STIs) including HIV/AIDS, unintended pregnancy, exploitation, stigma and discrimination, and violence [1–3]. FSWs are also ostracized by the community besides suffering legal and human rights violations thereby necessitating clandestine operations that often prevent them from accessing and/or using formal health services [4]. A qualitative study on the psychological stressors in the context of commercial sex in China showed FSWs face a host of stressors precipitated by poverty, unemployment, lack of social protection, violence from clients, and limited social support from peers and stable partners [5]. Another qualitative study conducted in Central Kenya identified three barriers to FSWs’ access to contraceptive services which comprise: unsupportive clinic infrastructure, long wait time, user fees, inconvenient operating hours and perceived compulsory HIV testing; discriminatory provider–client interactions, where participants believed negative and differential treatment from female and male staff members impacted FSWs’ willingness to seek medical services; and negative partner influences, including both nonpaying and paying partners [6].

All these vulnerabilities, coupled with women’s low status, repeated human rights violations, poor educational or economic opportunities and poor attitudes towards sex and sexuality, poor knowledge and access to modern contraceptive methods predispose women, and particularly FSWs, to a host of other STI risk factors [7–9]. The realization that key population groups such as sex workers are at increased risk of STIs led to the implementation of STI control widely adopted as a strategy to mitigate untoward effects of STIs/HIV [10]. Therefore, public health interventions targeting FSWs are designed to prevent STIs/HIV through correct and consistent condom use [10, 11]. Despite this, data suggests that unprotected sex and pregnancy are common among FSWs [12]. Unprotected sex further complicates the reproductive health needs of FSWs by predisposing them to abortion and other associated the risk factors. In Asia and Africa, for example, the prevalence of abortion among FSWs range between 22 and 86%, a pointer that FSWs’ pregnancies are ill attended [4]. A study among FSWs in Zambia showed that women who reported a lifetime pregnancy also had a history of unplanned pregnancy, depicting a large unmet need for contraception among FSWs [13]. Additionally, the strategies targeting FSWs remain ineffective by only targeting FSWs and failing to target their clients who are likely to be the decision makers in their sexual relations [14]. Evidence indicates that correct and consistent condom use may be complicated by the lack of autonomy to insist on condom use especially with steady and emotional partners, or through coercion by other clients who refuse to use condoms by promising to pay more or using violence [15, 16].

Since FSWs are at an increased risk to both unwanted pregnancy and sexually transmitted diseases, some have resorted to dual protection (simultaneous use of both condoms and female-controlled modern non-barrier methods), known to be an essential component of comprehensive reproductive health services [11, 13]. Approximately 67% of participants reported using a non-barrier method for contraception, but fewer than 20% of participants reported using both condoms and a non-barrier method. The study concluded that access to and use of dual protection is an essential component of comprehensive reproductive health services, particularly to women without current pregnancy intention [13].

FSWs need access to comprehensive SRH prevention measures [17]. Existing programs pay little attention to the broader sexual and reproductive health and rights of these women and often focus on HIV and other STIs prevention, care and treatment while neglecting the reproductive health needs, including awareness and access to a full range of family planning methods [15]. Also, these interventions have not fully embraced FSWs specific SRH needs like they have with women in the general population [1, 17]. Further, the challenges are augmented by discriminatory community values and norms, availability and access to the contraceptive methods suitable for FSWs [18]. This is mainly due to obstructive factors such as long waiting time, inconvenient waiting hours, user fees, and perceived HIV testing among other factors [17]. Unlike women in the general population, FSWs are prone to higher maternal morbidity and mortality risks because of exposure to risk factors associated with HIV-related mortality and unsafe abortion related deaths [19]. A study conducted in sub-Saharan Africa established that women who are engaged in commercial sex are at high risk of physical and sexual violence, unwanted pregnancy, and STIs [1]. The risk of violence further predisposes them to other social and health problems that hinder their access to SRH services which is a fundamental human right [20]. There are limited studies that have reported pregnancy desires and contraceptive use among FSWs, particularly in Kenya [15]. The available research, however, demonstrates that FSWs often want to avoid future pregnancies, despite higher rates of unplanned pregnancies and abortions compared to women in the general population [21, 22]. Although this is the case, they continue facing challenges in initiating and sustaining the use of more efficient contraceptive methods [15, 17, 23].

Access to contraception and reproductive health services remains a significant challenge for many sex workers. Studies suggest that many sex workers who did not want to become pregnant were not accessing a reliable contraceptive method often due to discrimination and fear, unfriendly health facility staff, and opening and closing time of services [24]. It is also worth noting that to a large extent, contraceptive uptake depends on individual perceptions, experiences, and ease of use [25]. Dual method approach is reported among FSWs mainly to help prevent unwanted pregnancy and diseases like STIs/HIV. Additionally, the dual method acts as a backup should a condom burst during sexual intercourse [11, 12]. The aim of this study is, therefore, to explore the experiences of FSWs with existing contraceptive methods while also considering the influence of clients who may act as barriers or offer opportunities for contraceptive use. It is anticipated that these findings will inform future interventions and access to services among FSWs and other key populations.

## Methods

The study was conducted from June to December 2008, within two districts - Naivasha and Changamwe – of Kenya’s former Rift Valley and Coast Provinces, respectively. These urban to semi-urban districts are known for having concentrated FSWs population, in part due to the port and tourist trade in the Coastal region in which Changamwe is part of, and to truck drivers, transport and seasonal workers in flower farms in Naivasha [15]. The locations are about 500 km apart; Naivasha is a vibrant town with a large migrant worker population attracted by its flower, transport and other industry. Other than being the second largest city in Kenya, Mombasa is a port city, trading center and popular tourist destination [26]. These two locations have long-running HIV and STI prevention programs for FSWs. These programs are run by various organizations providing targeted HIV and SRH services.

Women who reported current sex work, defined as ‘providing sexual services in exchange for money or other material compensation as part of an individual’s livelihood,’ and were 15–49 years were eligible for study participation. FSWs were recruited through local sex workers trained as HIV/AIDS peer educators and through snowball sampling [15, 26]. We utilized a targeted snowball across the two research sites (in Changamwe and in Naivasha) to recruit FSWs. FSWs serving organizations helped identify FSWs trained as HIV/AIDS peer educators. Initial contacts with FSWs varying in age, site of work, geographic location, and full or part time sex work status were made through trained peer educators already working in the study sites. These contacts were asked for referrals to other potential participants. Sex workers were screened and those found to meet study inclusion criteria were invited to participate in the study (survey/FGD). Approximately 6 FSWs did not meet the eligibility criteria. A total of eight Focus Group Discussions (FGDs) involved 10–12 participants (total 81). Participating women were grouped by similar age, site of recruitment and type of sex worker (full or part time) to enhance open discussion and reduce inhibitions among participants. The FGD guide addressed issues around FSWs health problems, work, health awareness of HIV, dynamics of their relationships with clients and contraceptive use.

The FGDs were conducted in the national language, Swahili, by a trained duo of a focus group moderator and a note taker. The same focus group research pair conducted all focus groups in both study sites to enhance consistency. FGDs were digitally recorded, uploaded to a laptop computer, transcribed verbatim, and translated from Swahili to English by the moderator and note taker. Transcriptions and translations were reviewed for quality by the interview team. The analysis team performed qualitative analyses with NVivo v. 7.0 (QSR International Pty Ltd) qualitative data analysis software. A content-driven theme approach was used for analytic review of the FGD data. Transcripts were read and re-read to identify recurrent themes and to develop a coding tree. Once all the transcripts were coded, memos and display matrices were developed to examine each code in detail for sub-themes, nuances, and patterns across the interviews [15, 26].

All FGDs took place at a neutral, confidential location secured by the research team in the study sites. Each participant was allocated a unique number, R1, R2…Rx to allow participant identification in each FGD. Additionally, the FGDs were labeled 1–4 in each location (Changamwe and Naivasha) and further the same criteria used to identify participant insights in the presentation of the findings. Cash compensation at a standard flat rate of Kshs 300 (approximately USD $4.29 at the time of data collection) was provided to all study participants upon arrival at the FGD site. This compensation was provided to reimburse participants’ transportation costs to the FGD site, and was approved by two institutional review boards, the Family Health International’s Protection of Human Rights Committee and the Kenyatta National Hospital Ethical Review Committee. At the close of the FGD, all participants received information on readily available service delivery points for Family Planning (FP) and HIV Counselling and Testing services [15, 26]. During the FGDs, the words ‘Kunga’ and ‘Sigalame’ were used to refer to FSW.

### Ethical considerations

Permission to conduct this study was obtained from Family Health International’s Protection of Human Subjects Committee (USA), the Kenyatta National Hospital/University of Nairobi - Ethical Review Committee (Kenya), and the National Council for Science and Technology (Government of Kenya) approved the study protocol. Consent was not sought from parents/guardians of participants aged below 18 years for this study because a sex worker is emancipated, sexually active and most often live alone. Verbal consent was provided by the study participants and waiver of signed informed consent was requested in accordance with 45 CFR 46. The study posed minimal risk to participants and the only record linking participants with the study would be the signed informed consent form. Oral informed consent with signature by the Research Assistant attesting to adherence to proper informed consent procedures and to the reception of the informed consent of the participant was therefore used in the place of signed informed consent forms. No identifying information was connected with the interviews or retained following the completion of the analysis.

## Results

The content analysis highlighted several themes concerning FSWs’ experiences with existing contraceptive methods, while also considering perceptions on male partners influence on contraceptive use. Our analysis covers the folling themes, practical experience with contraceptive use, opportunities and barriers to contraceptive use with different client types, dual protection and other barriers to contraceptive use. Many of these themes are underscored by gender power issues and reveal how sex work and contraceptive use in this setting are often areas of antagonism between FSWs and their clients.

### Contraceptive knowledge

In the narratives, some women said that they use contraceptives to protect themselves from pregnancy simply because they already have children. On the other hand, some FSWs had little or lacked idea on contraceptives. Either, this was due to ignorance or lack of appropriate information on how contraceptives work or where to access services. Whereas, some FSWs lacked time access services at the family planning clinic. Consequently, lack of appropriate information on contraceptives resulted into undesirable outcomes, where some women ended up having five to six pregnancies before seeking family planning services.

*“R2 if she practices family planning, her health together with that of her children will be good” FGD 4, Changamwe*

*“R2 I want to clarify the point, the truth is that most FSW’s do not like family planning” “I was talking about myself then, now we are discussing Kunga (FSW). I do not use condoms or any other family planning method, they are history for me. When will I feel the pleasure of sex!” FGD 2, Changamwe*

*“R6 I can say maybe it’s for pregnancy prevention, the three children are enough. But she will only use the condom with those who want to, and she will not have sex with those who do not want to use the condom. At least she will use the condom as she is least interested with family planning methods; the little that she gets at the time will help her take care of the three children” FGD 2, Naivasha*

*“M: no, I meant how important is pregnancy prevention* *R7: it is important because she has other children and she should know when she is supposed to get her periods so that she does not get another child and the others will not suffer” FGD 4, Changamwe*

### Opportunities and barriers to contraceptive use with different client types

Female sex workers provided their insights and experiences of using contraceptives by different client types. The narratives present opportunities and barriers to contraceptive use with casual clients, regular clients, and boyfriends or emotional partners.

#### Casual clients

Casual clients are one time or unfamiliar men who pay cash or other resources in exchange for sexual services. These types of clients are strangers whose primary aim is sexual pleasure and gratification and they do not have any emotional attachment with sex workers. Pregnancy prevention discussion with casual clients depend on different circumstances. But mostly, casual clients interest is sex and they do not care whether the sex worker becomes pregnant or not. Some casual clients use condoms to protect themselves against HIV and other STIs while others refuse to use condoms and may occasionally become aggressive when asked to use condoms. At other occasions, when the FSW insists on condom use, the casual client may decide to reduce pay because they believe that condoms reduce sexual pleasure.

*“R2 Sigalame (FSW) should also be careful; there are men who simply tear the condom. The man does not want to speak about it because he knows very well that you are going to refuse his suggestion, so he wants to do things that you are unaware of”. FGD 3, Changamwe*

*“R6 You may get a client who does not want to use any form of protection and since you have seen the money offered is a lot, you accept to have sex with him…” FGD 1, Naivasha*

*“R6…discussion about condom use is just never done…it depends with the money that a client wants to pay” FGD 2, Naivasha*

*“R8 (blowing her face with paper) and maybe I do not know him, we met on the road and I would not refuse to have sex with him because I need the money. It is unlikely for me to tell him about the pregnancy because I would not know where or how to get hold of him and even if he gave me a telephone number, telling him I was pregnant would not help me much!” FGD 1, Changamwe*

#### Regular clients

Some relationships progress from casual to more stable/trusted partnership because of familiarity and trust developed over time. Typically, in these type of relationships, an in-kind payment such as entertainment, child care or tours are a common feature in exchange for sex. The clients and sex workers communicate frequently and may agree on making visits to the sex worker’s home for sex or meet up at an agreed lodging area. Depending on the level of trust, the regular client may opt to use a condom or not. Overall, pregnancy prevention remains a priority and key consideration to the FSWs health and wellbeing. The partner may or may not provide support for pregnancy prevention. Generally though, avoidance of pregnancy is important to the woman because it gives her the opportunity to continue with her work and take care of her family and children’s needs.

*“R3 it is important for Sigalame (FSW) to go to an FP clinic to get her womb tied (tubal ligation), get pill or injection because this will even prevent her from getting pregnant by her regular client, whom she could even suggest to going for VCT and know their HIV status. I think that if she went for family planning, it would be good for her” FGD 1, Naivasha*

*“R4 maybe this is a regular client, somebody you are used to who has promised to pay good money for sex. You arrive at the guest house and turns against you by beating you up, refusing to use a condom and forcing you into sex. It would be so hard to complain especially at place where everyone knows you are there consensually. You are forced to carry on despite hurting” FGD 1, Changamwe*

#### Boyfriend or emotional partner

The boyfriend or the emotional partner can either be a live-in partner or not. This type of partner may be a client who has moved from being a casual client to a regular client and over time developed a strong emotional attachment with the FSW. Some of these relationships are strong because there is a child/children involved in the relationship. Sometimes, the boyfriend/emotional partner is aware of what the woman does to earn her money and choose to accept the situation. With this type of partnership, the woman continues with sex work to meet her needs owing to the financial need in her family.

*“R7 but even with an emotional partner who depends on you for upkeep, you will still have to go out and do sex work. There is no way you can tell your children that you do not have money to take care of them because you did not go to work, that you were counting days. An FSW is at work every day” FGD 3, Changamwe*

*“R5 I was saying that others could tell you to have that child and will even promise to take care of the rest, but in the end will say, “I did not send you to get pregnant, take care of it” so the problem still remains mine (hand on chest)” FGD 1, Changamwe*

*“R7 others will tell you to get pregnant and once the child is born will claim that it’s not his and say the child does not look like his brother or sister or even himself, which means he has already left you alone. Taking care of the child would be a problem for Sigalame as she is used to sex work for her livelihood” FGD 4, Naivasha*

Sometimes the unemployed boyfriend/emotional partner will encourage FSW to continue working to support the household. Even though the boyfriend/emotional partner knows that the FSW is involved in sex work, this does not bother him as long as she gets the money and provides for the family.

In the existing relationship with the emotional partner, the burden of taking care of children is solely on the FSW because the boyfriend may be unable to take up responsibility. For the emotional client, the FSW does not necessarily use a condom when having sex but may opt for other contraceptive methods based on her need. She might opt to use other methods such as the injectable, calendar or female condom as a family planning method. The FSW probably knows the HIV status of the live-in boyfriend or trusts him so they do not see the need to use condoms.

*“R7 She does not use any protection, but because she does not want to get pregnant and the man she is living in with wants her to, “how do I succeed in making this one pregnant?” it would force her to use her own condom to prevent pregnancy” FGD 3, Naivasha*

*“R4 it is very easy to manage an emotional partner and you cannot have sex with him every day anyway. If your period is very regular and are not using a condom with him it is easy to prevent pregnancy if you discuss it, he cannot refuse” FGD 1, Changamwe*

*“R3 our Sigalame (FSW) likes to count days if she has a boyfriend in the house, so she will count up to the date when she knows that if she had sex on that day, she would not get pregnant” FGD 4, Naivasha*

*“R8 even though she has different clients, there is one particular one she refers to as hers; and this one would not be using a condom all the time” FGD 2, Changamwe*

*“R2 but some Sigalame who live with their boyfriends in the house cannot prevent getting pregnant by him, maybe only those that she goes out to solicit” FGD 3, Naivasha*

*“R3 there is also another method that she could use; there is an FP method where if she got her period, will not be able to have sex with her boyfriend without a condom two weeks after” FGD 4, Changamwe*

*“R3 she makes them wear a condom, but she would not use a condom with the one in the house because they have been to a Voluntary Counseling and Testing {VCT} and know that they are HIV negative” FGD 3, Changamwe*

### Dual protection

The use of other contraceptive methods such as the injectable in addition to condoms was highly recommended by women to offer dual protection against pregnancy and STIs. To most women, the injectable or other methods are important because they can help prevent unwanted pregnancy when a condom breaks or the client intentionally tear them. Similarly, the use of pills was approved for the same reason as injectable.

*“R2 she will use a condom to prevent infections, but the injection would be to prevent pregnancy because of instances where the condom breaks, or a client who is cleverer and tears it” FGD 1, Naivasha*

*“R4 (trying to remember) she had said that (pointing at a participant) she should just use the injection and not the condom; but I am saying that whether one uses injection or pills, it is a must to use the condom because it prevents infections unless it broke or was torn by a client” FGD 2, Naivasha*

The female condom was recommended by some women who reported that it is a good method for protection from HIV and pregnancy because it can be used discreetly without the client's knowledge (Some FSWs serving organizations provide female condoms to FSWs in Mombasa and Naivasha). FSWs will most certainly use the female condom if they have self-awareness and are conscious about their health or don’t want to have more children.

*“R3 she will wear it before even going out there to look for clients’ maybe more than six hours earlier; if she gets one who does not want to use protection, she will be able to accept because she already has her own and this man will not know that” FGD 2, Changamwe*

### Other barriers to contraceptive use

It is advisable that FSWs use condoms correctly and consistently for STI/HIV prevention in addition to other more effective non-barrier contraceptive methods for pregnancy prevention. However, this may be a challenge to some FSWs given the difficulties they experience with non-barrier contraceptive methods. For instance, some FSWs intimated that side effects of contraceptives interfere with their engagement in sex trade. For instance, injectables were reported to be bad for business as they caused dizziness, nausea and continuous bleeding.

*“R7 There are also those who bleed all the time because of the injection. Once you start having sex, you bleed” FGD 2, Changamwe*

*“R2 Injection. Some bleed so much when they are on the injection. Or even when you are having sex, you find that you have been bleeding. That will also make clients run away from you” FGD 1, Changamwe*

*“R4 most Sigalame (FSW) do not like their period, will create problems during work. If it does not come the better* *M but do you know that a woman must get her period if she is not pregnant?* *R4 it is not a must if you use some of these FP methods, it does not come* *M like which ones?* *R1 injection” FGD 1, Naivasha*

*“R1 it really depends with someone’s body, something like the injection would affect people different, others hardly get their periods while some experience bleeding almost everyday” FGD 4, Changamwe*

Considering the pill, most FSW reported that this method was not popular with them because of the risk of forgetting to take a dose or they may be forced to take more pills to account for missed doses which may ultimately pose health risks.

*“R5 Even that family planning, let’s take for example the pill, Kunga (FSW) will not even have the time to take them, or she will simply forget to take them. Maybe she buys them every day and has a variety of packets in the house and does not even know which one to take” FGD 2, Changamwe*

*“R4 maybe you have the pills at home and have gone to Mtwapa (pointing) {Mtwapa is a town along the Mombasa-Malindi Highway} and was not able to come back for it the next day if you were forced by circumstances to stay there longer” FGD 4, Changamwe*

*“R7…he has asked you to stay with him for three days. If you have using pills and have left them at home, you will definitely get pregnant since you will not use a condom with him” FGD 2, Naivasha*

The challenges with an intrauterine device (IUD) or coil are related to the client feeling discomfort during sex and hence interfere with their business. These challenges coupled with the need to use a method that offers protection from STIs/HIV and unwanted pregnancy may result in condom use among the FSWs.

*“R3 she does not want to use the coil because you may feel it during sex and wonder what it could be” FGD 1, Naivasha*

*“R8 it’s not advisable to use the coil* *R3 it is very dangerous* *M why dangerous* *R3 because you go with this one, then another person like that (gesticulates)* *R7 and that is something which has been placed there {in the uterus}* *R3 and anyone can push it so she will have to be operated on* *NT how is it pushed?* *R7 it depends on the person you have been with* *R3 if the man is ‘big’” FGD 3, Naivasha*

On the contrary, use of the coil was not recommended by most women simply because of fear that it can come out during sex and may require a complicated medical procedure to insert. It seems FSW views of the coil is linked to poor knowledge of the device and anticipated interference during sexual intercourse.

*“R3 it will come out very fast and may even need an operation for it to be removed. So, anyone who does not have a husband” FGD 4, Changamwe*

*“R3 Sigalame cannot use a coil* *R2 because she ‘meets’ {has sex} with many people” FGD 2, Changamwe*

### Health facility-level barriers

Health facility-level barriers like having a mandatory HIV test may prevent FSW from accessing contraceptive related services. Most women fear being tested for HIV when they visit a family planning clinic. Some FSWs reported that they are forced to take an HIV test at the clinic, and therefore opt to visit private outlets such as pharmacies where they indicated that they receive good treatment. However, lack of friendly services prevents some women from accessing contraceptive methods.

*“R7 but some fear the injection because nowadays they are forced to test for HIV if you want to get the FP injection; and that is why when Sigalame (FSW) is tested and found to be HIV negative, she gets saved (salvation)” FGD 2, Changamwe*

*“R2 in my opinion I think that a private clinic will not give you anything that will or is going to harm you later on, they have to make sure that it is good for you” FGD 4, Changamwe*

*“M let us say she has been to a private clinic?”* *R5 she will feel good and has been treated well (gesticulating)* *M what about Municipal?* *R3 not satisfied, feel stigmatized* *R2 she will not feel good because she has had to queue and the doctor has hurried along so she will leave asking herself, “What exactly did I do over there?” FGD 2, Changamwe*

There is a perception that family planning will make one not to enjoy sex or be ‘sweet’ enough during sex, consequently, she will lose clients.

*“R2 Because family planning will spoil her ‘goods’ {sex] (pointing at herself)” FGD 1, Naivasha*

*“R6 Once they have seen it is [sex] not good, they will not come back” FGD 2, Changamwe*

Wait time and time spent at the health facility when accessing family planning services present an enormous challenge to some FSW. Some FSW narrated that they take long to access services and thereby risk losing clients when they are away at the family planning clinic. Moreover, there is stiff competition for clients, and no one wants to be left out in the search of new clients. Thus, to ward of competition from their peers, some FSW do not access contraception as they desire to be in business and attract clients.

*“*R6 *Kunga (FSW) lives in a guest house. We said, for example, she lives in Mwangeka guest house, and there are others living in the same guest house who are competing for the same clients. Maybe Kunga (FSW) wants to use protection; the others are fast ‘bamba fifty’ [cheap and do not use protection] and keep on getting clients. Kunga (FSW) will definitely stop thinking about contraception and follow suit” FGD 2, Changamwe*

## Discussion

This study makes a contribution by highlighting the experiences of female sex workers with contraceptives. We note that while some FSWs know that contraceptives are useful for pregnancy prevention, general knowledge remained poor with some resorting to abortion to terminate an unwanted pregnancy. Elsewhere, a study in China conducted among adolescent FSWs also found general sexual and reproductive health knowledge to be low, and while 98% reported not wanting the pregnancy, less than half (43%) reported consistent condom use with another 28% reporting current use of another contraceptive method [27]. A separate study examining contraceptive use among female entertainment sex workers in Cambodia found several factors to be linked to unwanted pregnancy such as the increase in a number of clients, inconsistent condom use, condom breakage and forced unprotected sex [28]. Elsewhere, unwanted pregnancy was more common among older married women who additionally had lower contraceptive knowledge [29].

The study also highlights the vulnerability of FSWs to unintended pregnancy or worse HIV/AIDS among those who have to balance between their livelihoods and pregnancy prevention with different types of sexual partners. In examining these FSWs’ contraceptive needs, it was clearly evident that on a daily basis these women were exposed to difficult situations that can have far-reaching implications on their overall health and well-being. In our analysis, we found that typically clients do not care much about contraceptive use – regardless of the partner type *–* most clients care less about using different contraceptive methods. Further, condom use for HIV/AIDS prevention was also difficult as some clients offer to pay more money to have unprotected sex while others turned violent against the women. This behavior by clients brings a lot of confusion to women. On the one hand, they have to make a difficult decision on whether or not to use condoms when enticed with a lot of money and on the other hand they are continually exposed to danger for proposing condom use to the clients. Nevertheless, it was common for women to describe pregnancies that occurred during sex work - commonly unintended.

Participants’ accounts of their contraceptive use (or non-use) with clients, also highlight the substantial diversity in women’s relationships with their clients. Whereas it was apparent that FSWs found it substantially difficult to discuss contraceptive use with casual clients, it appeared, however, that in ongoing relationships with regular clients, including boyfriend, lover, and emotional partner, there was consensus to use or not use contraceptives. Likewise, the extent to which women proposed contraceptive use to their clients or other sex partners varied dramatically by partner type. Women spoke about how their desire to use contraceptives with casual clients was in most cases dismissed by these clients whose interest is mainly sexual pleasure, and who sometimes forced them to have sex without any form of protection, from STIs/HIV. Although most women were open about discussing contraceptive use with their more familiar partners, most women revealed that most men deserted them when they learned that they were pregnant. For several women, getting pregnant and having children was a woman’s responsibility.

When participants described contraceptive use or pregnancy prevention, in most cases, the women either faced barriers to discussing contraceptive use with their partners or they had not used contraception consistently. Although most women seemed aware of the need to prevent pregnancy and were aware of other contraceptive methods, they clearly had difficulties in using them effectively with the different partner types due to violence; the lure of money; fear of losing them to their colleagues; or limited communication with their partners. Previous studies elsewhere in Sub-Saharan Africa demonstrate that interventions that sensitized male partners led to a significant increase in couple communication and a consequent increase in contraceptive use among couples [30]. Programs and providers that offer family planning services should, therefore, ensure that FSWs are empowered to use contraceptive methods, have access to contraception and that male partner are sensitized on the importance of contraception.

Consistent with previous studies among FSWs, inconsistent condom use was very common among participants. Compared to FSWs in similar settings as Kenya, women in our study face multiple barriers in ensuring that they have protected sex by having their clients use condoms [5]. Based on their accounts, most participants wished to use condoms with casual clients, however, convincing their clients to use condoms was not always easy. Although there was a notable concern from the women of fear of being infected with HIV or other STIs, some of the clients seemed not to share these concerns. Most of these clients ultimately forced or lured the women to have unprotected sex.

Most of the time, sex with the emotional partner or boyfriend was without condom use as many FSWs reported a preference for the injection or calendar method with these partners. Similar findings have also been reported in a study conducted in Nyanza where FSWs reported unprotected sex with their regular or romantic partners. Female sex workers interviewed in Kibera in Nairobi also reported not using condoms with their intimate partners as this was a sign of intimacy and trust [31]. Overall, despite reported use of other contraceptive methods, there was over-reliance on condoms which offer dual protection.

Dual protection was a valuable tool for prevention of unwanted pregnancy and STIs/HIV. Some FSWs reported using condoms and other forms of contraceptives such as injectables and pills to offer dual protection. While condoms are effective at preventing STIs/HIV, they may not be very effective in preventing unwanted pregnancy. On the other hand, non-barrier contraceptive methods do not offer protection against STIs/HIV hence the need to use both to offer dual protection [11]. Fear of condom breaking was also reported as a motivation for dual protection to offer protection against unwanted pregnancy. On the other hand, it was worth noting that dual protection was not common with the emotional partner or boyfriend with whom the FSW reported to use either the calendar method or injectables and their relationship is based on trust. Elsewhere, use of non-barrier methods and condoms was found to be less among FSWs and their non-commercial, often more intimate partners [11]. Additionally, a study in Gulu, northern Uganda reported low dual contraceptive use as a result of police presence which led to rushed negotiations with clients thereby increasing the FSW risk to STIs/HIV and unwanted pregnancy [32]. The use of female condoms was also reported to offer protection, especially with clients who refused to use the male condom. This was mainly used by FSW who were concerned about STIs/HIV and unwanted pregnancy. The female condom has been found to offer dual protection from both STIs/HIV and unplanned pregnancy; it also acts as a tool for women’s empowerment.

Reported barriers to contraceptive uptake among FSWs include side effects which interfere with their business of sex trade such as continuous bleeding, dizziness, and nausea for the injectables. Other barriers were those related to access to the services which included fear of getting tested for HIV whenever they visited family planning clinics, competition in clinic time and time for clients, among other barriers. To increase family planning uptake among FSW in Cambodia, the government and NGOs provide free and friendly sexual and reproductive health services, despite this, some FSW still reported barriers such as discrimination by providers thereby making them resort to using of private providers [28].

## Conclusion

Program implementers should consider working with providers to minimize barriers as FSWs face substantial barriers to making decisions around contraceptive access and use. There are also myths and misconceptions among FSWs around family planning use which sometimes they say will spoil her ‘goods’ and eventually make them lose clients. We, therefore, recommend the introduction of counseling services to provide information on the benefits of non-barrier contraceptive methods and additional support services to manage side effects arising from their use. Moreover, more family planning distribution points within the community especially those targeting priority groups such as sex workers will help increase access and ultimately increase contraceptive uptake among FSWs.

## Abbreviations

CFR: Code of Federal Regulations
FGD: Focus Group Discussion
FHI: Family Health International
FP: Family Planning
FSWs: Female Sex Workers
HIV: Human Immunodeficiency Virus
HIV/AIDS: Human Immunodeficiency Virus/ Acquired Immuno-Deficiency Syndrome
IUD: Intrauterine Device
NGOs: Non-Governmental Organizations
SRH: Sexual and Reproductive Health
STIs: Sexually Transmitted Infections
STIs/HIV: Sexually Transmitted Infections/ Human Immunodeficiency Virus
USAID: US Agency for International Development
VCT: Voluntary Counseling and Testing
WHO: World Health Organization
